# Cryoprotective Effect of NADES on Frozen-Thawed Mirror Carp Surimi in Terms of Oxidative Denaturation, Structural Properties, and Thermal Stability of Myofibrillar Proteins

**DOI:** 10.3390/foods12193530

**Published:** 2023-09-22

**Authors:** Haijing Li, Qian Wang, Wenxin Li, Xiufang Xia

**Affiliations:** College of Food Science, Northeast Agricultural University, Harbin 150030, China; lhjing0131@163.com (H.L.); qianwang0201@163.com (Q.W.); 19845916914@163.com (W.L.)

**Keywords:** natural deep eutectic solvents, frozen-thawed surimi, myofibrillar protein, oxidative denaturation, structure stability

## Abstract

Quality degradation due to the formation and growth of ice crystals caused by temperature fluctuations during storage, transportation, or retailing is a common problem in frozen surimi. While commercial antifreeze is used as an ingredient in frozen surimi, its high sweetness does not meet the contemporary consumer demand for low sugar and low calories. Therefore, the development of new green antifreeze agents to achieve an enhanced frozen-thawed stability of surimi has received more attention. The aim of this study was to develop a cryoprotectant (a mixture of citric acid and trehalose) to enhance the frozen-thawed stability of surimi by inhibiting the oxidative denaturation and structural changes of frozen-thawed mirror carp (*Cyprinus carpio* L.) surimi myofibrillar protein (MP). The results showed that the amounts of free amine, sulfhydryl, α-helix, intrinsic fluorescence intensity, and thermal stability in the control significantly decreased after five F-T cycles, while the Schiff base fluorescence intensity, amounts of disulfide bonds and surface hydrophobicity significantly increased (*p* < 0.05). Compared to sucrose + sorbitol (SS), the natural deep eutectic solvents (NADES) effectively inhibited protein oxidation. After five F-T cycles, the α-helix content and Ca^2+^-ATPase activity of the NADES samples were 4.32% and 80.0%, respectively, higher, and the carbonyl content was 17.4% lower than those of the control. These observations indicate that NADES could inhibit oxidative denaturation and enhance the structural stability of MP.

## 1. Introduction

Surimi constitutes a wet, frozen concentrate of myofibrillar protein (MP) extracted from marine or freshwater fish and is low in fat and cholesterol [1]. With the continuous depletion of high-quality marine resources, the importance of freshwater fish is gradually increasing. As a freshwater fish of commercial value in northeastern China, mirror carp has high protein and low fat content. Currently, mirror carp is mainly sold in fresh live form with a single processing method, and the corresponding post-processing technology used in the north of China is outdated [2]. In order to maximally maintain the sensory properties and nutritional value of mirror carp flesh, processing into surimi is preferred in commercial production. However, raw surimi does not meet the market demand due to its concentrated release period and the requirements for effective storage and transportation methods to maintain its high quality.

Freezing is a widely used and low-cost cryopreservation method that can inhibit microbial reproduction and reduce enzymatic activity [3]. However, repeated freezing and thawing caused by imperfect cold chain technology can produce large and unevenly distributed ice crystals during storage and circulation [4]. Ice-crystal-impacted muscle tissue can accelerate the release of reactive oxygen radicals and their attack on MP, exacerbating the oxidation and denaturation of the protein [5]. The surface groups of myosin with the α-helical structure are ionized and aggregated due to water migration and conversion to ice crystals. At the same time, the sequential solidification (freezing) of free water and bound water changes their ionic strength, transforming the three-dimensional structure of the protein from a highly hydrated folded state (steady state) to an unfolded state [6]. In the unfolded state, the exposed non-polar groups generate hydrophobic interactions, hydrogen bonds, disulfide bonds, and ionic bonds, leading to protein conformational rearrangements and aggregation. In addition, muscle tissue punctured by ice crystals can release proteases, whose hydrolysis leads to the disruption of the structure of MP (especially myosin and actin), a skeletal protein, and the formation of small subunits, which can lead to a decrease in thermal stability [7]. Therefore, it is essential for the quality of frozen surimi to reduce the mechanical damage of ice crystals during freezing and thawing.

Recently, studies on controlling the oxidative denaturation of surimi proteins by inhibiting the growth of ice crystals during freezing have concentrated on physical field-assisted freezing techniques [8] and antifreeze agents [9]. However, the physical field-assisted freezing techniques have some limitations, such as complexity and the high cost of equipment. Therefore, antifreeze agents as a raw material for the production of frozen surimi have research significance and prospects. Currently, commercial antifreeze agents, such as polysaccharides, are limited by their high-calorie content, high sweetness, and protein cross-linking obstruction, which results in the inhomogeneous microstructure of surimi. The colored properties of polyphenols can reduce the sensory properties of surimi [10]. The intake of polyphosphates should be rigorously managed because their overuse can lead to the inhibition of calcium absorption [11]. Antifreeze proteins/peptides [12] and protein hydrolysates [13] are widely studied for their favorable antifreeze activity, yet their prohibitive cost, low yield, and harsh production conditions limit their commercial applications. In nature, some cold-blooded animals can produce partially non-toxic biodegradable metabolites that form hydrogen bonds in a certain molar ratio to create antifreeze systems with high viscosity and a supramolecular network, which are called natural deep eutectic solvents (NADES). This system limits the free movement and arrangement of water molecules, enabling the reduction in ice crystal production. Additionally, the freezing point of the system is decreased by taking advantage of the charge delocalization effect, which keeps it in a glassy state at freezing temperature [14]. Currently, 135 binary NADES based on primary metabolites have been reported, which can further form multicomponent eutectic solvents (e.g., quaternary or ternary NADES) [15]. Castro et al. [16] first proposed the possibility of eutectic solvents as a new non-toxic antifreeze agent and verified the physical stability and toxicity of the eutectic system based on the formation of trehalose and glycerol. On this basis, Jesus et al. [17] combined hydrogen bond donors and hydrogen bond acceptors such as trehalose, glycerol, betaine, glucose, and sorbitol to construct and obtain 18 groups of NADES, and further verified their physical stability and toxicity. With the ongoing research, the application of NADES in muscle foods is at a beginning and emerging stage. Tian et al. [18] prepared four edible NADES [(Pro:Glc (5:3), Pro:Glc (1:1), Pro:Sor (1:1), and urea:Glc:CaCl_2_ (3:6:1)] for the preservation of frozen chicken breasts via interfacial antifreeze. Additionally, Du et al. [19] (our previous study) prepared edible NADES from food-grade citric acid and trehalose for the antifreeze preservation of frozen surimi. At present, NADES is mainly applied to the cryoprotection of muscle foods in the form of a contact interface [18], and its direct application in the form of antifreeze for the cryoprotection of surimi is in its infancy. 

Citric acid is a widespread natural antioxidant that is permitted by the Codex GSFA as a food additive for frozen fish [20]. Trehalose is a non-reducing disaccharide from nature that is extremely stable and moderately sweet (45% of the sweetness of sucrose), making it an ingredient that meets the modern concept of a healthy diet [1]. Meanwhile, Choi et al. [21] explored the effects of NADES prepared from citric acid and trehalose on cellular metabolism and physiology. Furthermore, Ferreira et al. [22] evaluated the toxicity and antioxidant properties of homozygous NADES on zebrafish, verifying its safety and usability in aquatic products. The inhibitory effect of citric acid and trehalose-based NADES on the quality deterioration of surimi has been preliminarily verified by our team [19]. On this basis, the objective of this study was to prepare a cryoprotectant (a mixture of citric acid and alginate) via hot mixing, and it was added to surimi using a direct mixing method to investigate its cryoprotective mechanism on the quality deterioration of frozen-thawed mirror carp surimi in terms of the oxidative denaturation, structure, and thermal stability of MP. Therefore, we hypothesized about NADES, which is based on citric acid and trehalose, is non-toxic and has good antioxidant properties, combined with its strong hydrogen bonding network. Can it play a cryoprotective role in frozen surimi by reducing the water flow and inhibiting the mechanical damage of ice crystals? The aim was to provide a theoretical basis for the development and application of novel and green antifreeze agents and to provide new insights into improving the quality of frozen freshwater surimi.

## 2. Materials and Methods

### 2.1. Sample Preparation

#### 2.1.1. NADES

The NADES was prepared by heating and stirring the mixture of citric acid monohydrate (98% purity, CAS 5949-29-1) and D-trehalose dihydrate (99% purity, CAS 6138-23-4) at 60 °C in a molar ratio of 2:1. The mixture was heated on a C-MAG HS10 heating plate (IKA GmbH, Staufen, Germany) and stirred (100 rpm) until a clear and transparent solution was formed. Successful preparation of the sample can be determined by its ability to maintain stability after being cooled down to the standard room temperature of 25 °C. Extensive hydrogen bonding networks and electrostatic and van der Waals interactions between the NADES components resulted in the limited mass transfer of free substances in the system. The high viscosity of NADES can hinder its practical application, and to maximize its high-quality physicochemical, antifreeze, and recrystallization properties [19], 10% (*v*/*v*) distilled water was added to the NADES system for subsequent measurements.

#### 2.1.2. F-T Surimi and MP Extraction

Twelve live mirror carp (1200 ± 20 g) were obtained and handled for the preparation and processing of surimi based on previous research in the lab by Du et al. [19]. There were three treatments: control (no cryoprotectant), 4% sorbitol + 4% sucrose (SS), and 4% (*w*/*w*) NADES (clear and transparent liquids with 10% moisture content). They were frozen at −18 °C (7 d) and then thawed at 4 °C (12 h), which was the first F-T cycle (F_1_). Further, the above F-T process was repeated three and five times (F_3_ and F_5_). The extraction and content measurements of MP were performed according to the method reported by Li et al. [23]. Surimi (40 g) was mixed with 4 volumes of extraction solution and homogenized using a tissue masher (DS-200, Ronghua Instrumental Manufacturing Co., Ltd., Changzhou, China) for 60 s. The resulting homogenate was centrifuged using a refrigerated centrifuge (Xiangyi Laboratory Instrument Co., Ltd., Changsha, China) at 6500× *g* at 4 °C for 15 min. The precipitate was extracted twice with 4 volumes of the extraction solution as described above and the process was repeated. The precipitate was washed twice with a 4-fold volume of pre-cooled crushing buffer (0.1 M NaCl), as described above. The precipitate was then redissolved with a 4-fold volume of pre-cooled crushing buffer and filtered through 4 layers of gauze. After filtration, the suspension was centrifuged under the above conditions to obtain the MP precipitate. Lastly, MP concentration was measured using the Biuret method. The standard curve was y = 0.0525x + 0.0036 (R^2^ = 0.9994).

### 2.2. Determination of MP Oxidation and Denaturation

#### 2.2.1. Schiff Base Fluorescence Intensity

The determination method was based on that reported by Li et al. [24]. The flesh (1.0 g) was spiked with 10.0 mL sodium phosphate buffer and subjected to homogenization for 30 s. The supernatant (1.0 mL) was diluted with 20.0 mL sodium phosphate buffer after centrifugation, and then the fluorescence emission spectra of the samples at 380~600 nm was recorded using an F-7100 fluorescence spectrophotometer (Hitachi Co., Tokyo, Japan). The excitation wavelength was 360 nm, and the results were expressed as fluorescence intensity.

#### 2.2.2. Carbonyl Content

The protein samples were treated with DNPH and HCl, respectively, and the treated solutions were subjected to a nuance spectrum, where HCl was the control. The standard of the assay system was BSA in guanidine, and spectroscopic measurements of the control samples were conducted. The value at 280 nm was the protein concentration at this point. The measurement results were expressed as nmol of DNPH per mg of protein absorption. The light absorption of the protein hydrazone at 370 nm (21.0 mmol/L·cm^−1^) could be used as a reference.

#### 2.2.3. Ca^2+^-ATPase Activity

The system containing NaCl (0.5 mol/L), 5 mM CaCl_2_ (5 mmol/L), ATP (1 mmol/L), trimellitate (pH 7.0, 25 mmol/L), and protein (6 mg/mL) was reacted at 37 °C for 10 min, and the reaction was ended by the addition of HCIO_4_ at a final concentration of 5%. The supernatant was then centrifuged (6000 rpm, 10 min) and the release of inorganic phosphate was determined by the phosphomolybdate method.

#### 2.2.4. Surface Hydrophobicity

The surface hydrophobicity of the MP was calculated according to Li et al. [25]. The concentration of MP was adjusted to 1.0 g/L with phosphate buffer (20 mM, pH 6.0), and then 1 mL of MP was mixed with 200 μL of bromophenol blue (BPB) and shaken for 10 min. After centrifugation at 2000× g for 15 min, 0.4 mL of supernatant was mixed with 3.6 mL of phosphate buffer, and the absorbance was measured at 595 nm using a UV–visible spectrophotometer (UV-721, Shanghai Precision Instrument Co., Ltd., Shanghai, China). The calculation formula is as follows:

(1) $$\mathrm{BPB}\;\mathrm{bound}\;\left(\mu g\right)=200\times \;(\frac{A_{\mathrm{blank}}-A_{\mathrm{sample}}}{A_{\mathrm{blank}}})$$

### 2.3. Determination of MP Structure Properties

#### 2.3.1. Amino Acid Residue Side-Chain Groups

Free amino groups and total and reactive sulfhydryl groups were assayed following the method of Du et al. [2], and the disulfide bond content was determined according to the method reported by Chen et al. [9]. The details of the experimental steps are as follows.

The samples were mixed thoroughly with 2.0 mg/mL MP and ortho-phthaldialdehyde (OPA) at 1:20 (*v*/*v*) and placed at room temperature for 2 min. The absorbance values at 340 nm were measured using an ultraviolet–visible spectrophotometer (Runqee Instrument Technology Co., Ltd., Beijing, China).

Further, 2.0 mg/mL MP was blended with tris-glycine buffer at 1:8 (*v*/*v*) and centrifuged at 10,000 rpm for 15 min. The supernatant was mixed with Ellman’s reagent (10 mmol/L DTNB) at 9:1 (*v*/*v*). After the reaction for 30 min, the samples were placed on a spectrophotometer, and the absorbance values at 412 nm were measured. The content of reactive sulfhydryl was determined in the same manner as for that of total sulfhydryl. The MP solution (1.0 mg/mL) was mixed with a 2-nitro-5-thiosulfobenzoate assay solution at 30:1 (*v*/*v*), and the reaction was conducted at room temperature for 25 min. The absorbance values were also measured at 412 nm. The disulfide bond content was calculated as half the difference between the total sulfhydryl and active sulfhydryl contents.

#### 2.3.2. Secondary and Tertiary Structure

The secondary and tertiary structures as well as the parameters of the MP were determined according to the method reported by Du et al. [2]. Two MP samples (0.2 mg/mL and 0.1 mg/mL) were placed on a Jasco J-815 spectropolarimeter (Jasco Corp., Tokyo, Japan) and an F-4500 fluorescence spectropolarimeter (Hitachi Co., Ltd., Tokyo, Japan), respectively, for the measurement of secondary and tertiary structures.

### 2.4. Thermal Stability

The thermal stability of surimi MP was assayed using a differential scanning calorimeter (DSC, NETZSCH-Gerätebau GmbH, Selb, Germany), as detailed in Du et al. [26]. Temperature was calibrated using indium thermography. The samples (8–12 mg) were sealed in standard aluminum trays, and the temperature was raised from 25 °C to 90 °C. The scanning rate was 5 °C/min. The control was an empty aluminum crucible.

### 2.5. Statistical Analysis

The experiment was repeated three times, with a minimum of three parallel samples measured in each independent experiment, and the obtained data are presented as mean ± standard deviation (SD). Data were analyzed through analysis of variance (ANOVA) using IBM SPSS 25 software, which underwent descriptive, fixed, and random effects and homogeneity-of-variance tests. The statistical significance of the data was verified using Duncan’s test (*p* < 0.05).

## 3. Results and Discussion

### 3.1. The Inhibition of NADES on MP Oxidation and Denaturation of Frozen-Thawed Mirror Carp Surimi

#### 3.1.1. The Inhibition of NADES on the Schiff base Fluorescence Intensity of Frozen-Thawed Mirror Carp Surimi

A Schiff base is an organic compound containing a C=N structure generated by the reaction of the free amino group from a protein with the aldehyde group in reducing sugar, reflecting the degree of oxidation of the protein [27]. Large amounts of reactive oxygen radicals can cause protein oxidative damage. The initiation point of the protein oxidation radical chain reaction is the production of alkyl radicals (C·) from reactive oxygen species (ROS)-attacking proteins. During frozen storage, oxygen promotes the conversion of C· to peroxyl radicals (COO·) [8]. Ice crystals puncture the cell membrane, releasing catalytic metal ions and increasing the concentration of pro-oxidants in the unfrozen phase around the protein molecule, which promotes the production of alkyl peroxides (COOH), alkoxy radicals (CO·), and hydroxyl derivatives (COH) [28]. Protein oxidation mainly consists of amino acid side chain reactions, fragmentation, and covalent cross-linking. Protein carbonylation is the most representative. Protein carbonylation consists of four aspects, including the direct oxidation of acid side chains (Michael adducts), non-enzymatic glycosylation (Schiff bases), the oxidative cleavage of peptide backbones via the α-amidation pathway or glutamyl side-chain oxidation, and covalent binding to nonprotein carbonyl compounds [29].

**Figure 1:**
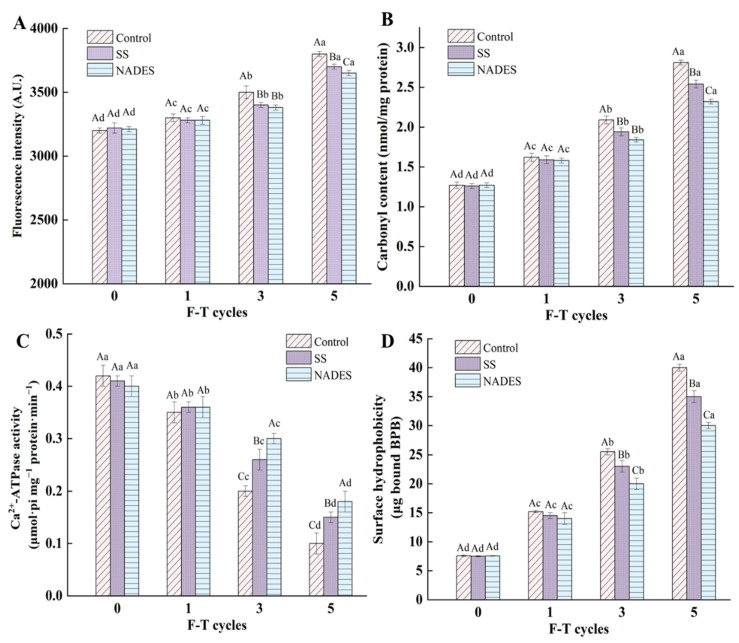
Effect of NADES on the Schiff base fluorescence intensity (**A**), carbonyl content (**B**), Ca^2+^-ATPase activity (**C**), and surface hydrophobicity (**D**) of MP from frozen-thawed surimi. The means at the same freeze–thaw cycles with different uppercase letters (A–C) differ significantly (*p* < 0.05); the means at the same NADES concentration with different lowercase letters (a–d) differ significantly (*p* < 0.05).

As observed in Figure 1A, the Schiff base fluorescence intensity of all samples increased when increasing the number of F-T treatments, suggesting that F-T treatments deepened the degree of MP oxidation. After five F-T treatments, the fluorescence intensity of the three groups of samples increased by 18.75% (control), 14.91% (SS), and 13.71% (NADES) compared with that of fresh surimi MP. This is probably due to the fact that muscle tissue punctured by ice crystals during the F-T cycles releases proteases that lead to protein degradation, which provides carbonyl and free amino groups, which promote Schiff base formation [24]. It is worth mentioning that the fluorescence intensity of the NADES group during the F-T process was lower than that of the control and SS groups. This phenomenon occurred because the strong hydrogen bonding network of NADES inhibits water movement and reduces the generation of ice crystals, which reduces the damage of ice crystals to the MP structure and inhibits the unfolding of the protein and the exposure of side-chain motifs [19]. In the case of Lys, the formation of Schiff bases is reduced by decreasing the exposed Lys, since Lys is the reactant for Schiff base formation.

#### 3.1.2. Inhibition of NADES on the Carbonyl Content of Frozen-Thawed Mirror Carp Surimi

Carbonylation is a representative reaction of protein oxidation and is indispensable to the spoilage of aquatic products [30]. The inhibitory effect of the NADES on the increasing MP oxidation in the F-T surimi is shown in Figure 1B. The carbonyl content of all samples increased with the number of F-T treatments, demonstrating the deepening of MP oxidation. During a complete freezing and thawing process, the conversion of liquid water to solid ice during the freezing phase and the growth of ice crystals and recrystallization during the frozen storage phase can generate huge ice crystals. This huge ice crystal formation can damage muscle cells and eventually lead to the release of various pro-oxidizing substances such as heme iron (approximately 9.9 mg/kg in surimi) and ROS [2]. In addition, during the freezing and storage of the frozen samples, the formation of ice crystals can cause an imbalance of intra- and extracellular osmotic pressure during the thawing phase, accelerating the loss of moisture, which accelerates the oxidative denaturation of MP against a background of solution effects [31]. However, the increase in the carbonyl content was not the same in the different treatment groups, with the highest increase in the control group samples, followed by the SS group samples, and the lowest increase in the NADES group samples. SS can encapsulate proteins through ionic or hydrogen bonds to provide protection [32]. The stronger antioxidant capacity of the NADES compared with SS may be based on its strong protective effect on the muscle tissue and the presence of citric acid, an antioxidant, in its composition. During frozen storage, the steric and secondary structures of proteins tend to disintegrate and the internal reactive groups are exposed, resulting in the formation of carbonyl and disthionyl groups and the reduction in free sulfhydryl groups, leading to protein oxidation, while the presence of citric acid inhibits this transformation [33]. Yu et al. [34] found that citric acid acts as an antioxidant that could inhibit oxidation by scavenging free radicals around protein particles. Therefore, the addition of citric acid can freeze storage-induced protein oxidation problems, thereby improving oxidative stability. In addition, the physical kinetic process of ice crystal formation in the frozen state includes crystallization (nucleation and growth) and recrystallization, which involves a dynamic water–ice transition. NADES prepared with citric acid and trehalose has a huge hydrogen bonding network system, and the presence of NADES inhibits the free movement of water and reduces the formation of ice crystals and volume extension, thus reducing the mechanical damage of ice crystals, decreasing the cryo-denaturation and structural deterioration of proteins, which suppresses the exposure of the internal reactive groups, and consequently inhibiting the formation of carbonyls [14,19]. 

#### 3.1.3. Inhibition of NADES on Ca^2+^-ATPase Activity of Frozen-Thawed Mirror Carp Surimi

**Figure 2:**
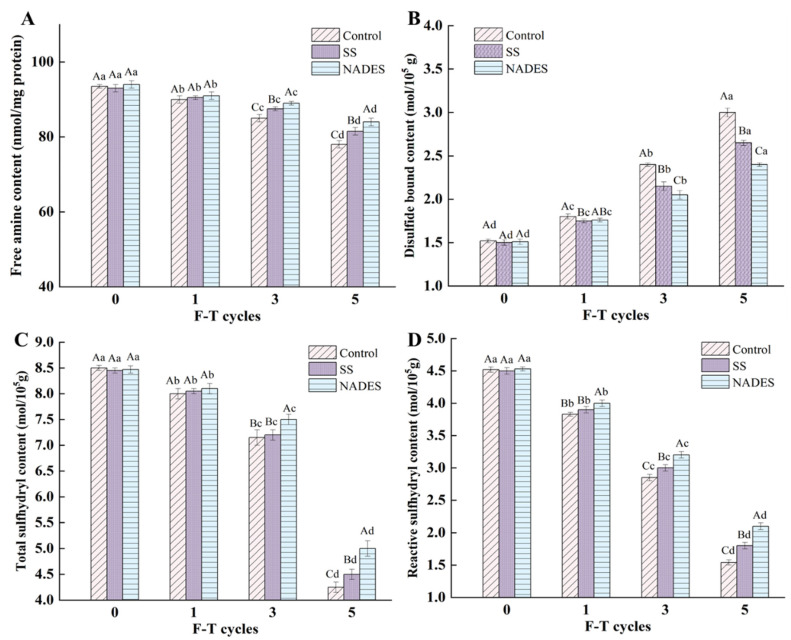
Effect of NADES on the free amine content (**A**), disulfide bond content (**B**), total sulfhydryl (**C**) and reactive sulfhydryl content (**D**) of MP from frozen-thawed surimi. The means at the same freeze–thaw cycles with different uppercase letters (A–C) differ significantly (*p* < 0.05); the means at the same NADES concentration with different lowercase letters (a–d) differ significantly (*p* < 0.05).

Ca^2+^-ATPase activity can be used as an indicator to evaluate the degree of MP denaturation. More precisely, Ca^2+^-ATPase activity can reflect the integrity of the molecular structure of myosin well because the globular head of myosin contains sulfhydryl groups that contribute to the Ca^2+^-ATPase activity [1]. The Ca^2+^-ATPase activities of the control group samples were 0.42 (fresh), 0.35 (F_1_), 0.20 (F_3_), and 0.10 (μmol·pi/mg·pro/min) (F_5_) (Figure 1C). The mechanical damage caused by ice crystals during freezing and the increase in ionic strength due to water loss during thawing exacerbate the conformational changes in myosin heads, exposing the sulfhydryl groups to oxidative denaturation and aggregation. In addition, protein rearrangement induced by protein–molecule interactions is another reason for the decrease in the Ca^2+^-ATPase activity [35]. The continuous decrease in total sulfhydryl and reactive sulfhydryl contents during the F-T phase also validates the myosin denaturation and the decrease in Ca^2+^-ATPase activity (Figure 2C,D). The SS and NADES group samples exhibited higher Ca^2+^-ATPase activity compared to the control group sample for the same number of F-T cycles; that is, after five F-T treatments, the Ca^2+^-ATPase activities for the control, SS, and NADES samples were 0.10, 0.15, and 0.18 (μmol·pi/mg·pro/min), respectively. Zhou et al. [1] demonstrated the cryoprotective effect of SS on the Ca^2+^-ATPase activity of surimi. The higher value of the Ca^2+^-ATPase activity of the NADES group sample compared to that of the SS group sample indicated that the NADES was more effective in inhibiting the decrease in the Ca^2+^-ATPase activity. This is attributed to the fact that the hydrogen bonding network system of NADES effectively inhibits water movement and reduces the generation of ice crystals, reducing the mechanical damage of ice crystals to muscle tissues [36], thus inhibiting conformational changes and the denaturation of myosin head and consequently stabilizing the Ca^2+^-ATPase activity.

#### 3.1.4. Inhibition of NADES on Surface Hydrophobicity of Frozen-Thawed Mirror Carp Surimi

Proteins in their natural state exist in a solution where their hydrophilic side chains face the water while their hydrophobic side chains face inward. Surface hydrophobicity can reflect the extent to which the hydrophobic amino acid residues located inside are exposed to the protein surface, and the degree of protein denaturation is reflected by the amount of bound bromophenol blue [37]. The surface hydrophobicity of all samples increased after the F-T treatments (Figure 1D). The F-T process generates a large number of ice crystals, and the mechanical damage to the muscle tissue by ice crystals exacerbates the degree of protein oxidation as well as conformational changes. In particular, the large ice crystals formed during the freeze–thaw process altered the tertiary structure of the MP, unfolding the protein structure and exposing more internal hydrophobic residues for binding to bromophenol blue [38]. When surface hydrophobicity increases, MP undergoes intermolecular aggregation, which ultimately leads to the irreversible denaturation of MP [39]. Different types of antifreeze agents have different inhibitory effects on the increase of MP surface hydrophobicity after experiencing freezing. The superior effect of the NADES over SS was demonstrated by the lower surface hydrophobicity value of MP after five F-T cycles. The strong hydrogen bonding network of NADES can inhibit water flow and reduce the generation of ice crystals, which reduces the damage of ice crystals to the MP structure, thus inhibiting protein unfolding and the exposure of hydrophobic amino acid residues [19]. Moreover, Sava et al. [40] showed that the oxidation of sulfhydryl groups also increased the surface hydrophobicity of proteins. The present study showed that NADES could protect sulfhydryl groups from oxidation to a certain extent (Section 3.2.1), thus delaying the increase in surface hydrophobicity. This phenomenon demonstrates the excellent role of NADES in inhibiting the freezing denaturation of MP, and relatively good cryoprotective ability can be obtained with a low addition.

### 3.2. Effect of NADES on MP Structure Properties of Frozen-Thawed Mirror Carp Surimi

#### 3.2.1. Effect of NADES on MP Amino Acid Residue Side-Chain Groups of Frozen-Thawed Mirror Carp Surimi

During the processing and storage of surimi, the protein side-chain structure, which contains various amino acids with free amines (proline, lysine, threonine, and arginine), tends to be disrupted due to temperature fluctuations that trigger repeated freezing and thawing. The change in protein conformation (unfolding) exposes certain amino acid residues, which in the presence of free radicals produce carbonyl groups, leading to enhanced oxidation and adversely affecting the quality of surimi. Therefore, the free amine content can be used as a reference to evaluate the changes in the amino acid side-chain groups of the MP during F-T cycles [37]. The free amine content of the control sample decreased from 93.5 (nmol/mg pro) (fresh) to 78.0 (nmol/mg pro) (F_5_) (Figure 2A), which was attributed to the disruption of the protein side-chain structure by ice crystals as well as the leaching effect of the drip loss or thawing loss, resulting in the loss of some nutrients (e.g., small molecule peptides and free amino acids) from surimi with water [41]. The e-NH_2_ group represented by lysine residues was easily oxidized to carbonyl derivatives in the presence of reactive oxygen radicals and metal ions, which then reacted with the available -NH_2_ groups, leading to a continuous decrease in free amine content [42]. It is noteworthy that the addition of cryoprotectants is useful for maintaining the stability of the amino acid residue side-chain structure. After five F-T cycles, the SS and NADES group samples were 4.49% and 7.69% higher than the control group samples, respectively. The free hydroxyl group of SS can bind to water molecules, which reduces the formation of ice crystals [43]. In contrast, the ability of the NADES to inhibit the growth of ice crystals and the protection of the amino acids side-chain groups appeared to be more effective. The strong hydrogen bonding network system established between the components of the NADES can efficiently trap the water migrating outside the muscle cells owing to the puncture of ice crystals [44].

Sulfhydryl groups—the most reactive functional group in the surimi protein—are mainly distributed in the head of the myosin and play an irreplaceable role in maintaining the structure of the protein [37]. Zhang et al. [45] reported that sulfhydryl groups belong to weak secondary bonds and maintain the tertiary structure of the protein, and changes in sulfhydryl content also can reflect the alteration degree of tertiary and quaternary structure. Multiple F-T treatments decreased the total sulfhydryl and reactive sulfhydryl content in all samples, as well as increasing the disulfide bonds (Figure 2B–D). The growth of ice crystals resulting from the F-T treatments disrupted the intact structure of MP, leading to structural unfolding as well as the exposure of side-chain cysteine residues. At this time, the sulfhydryl groups were easily converted to disulfide bonds by a reactive oxygen radical attack. Further, the presence of disulfide bonds was the main reason for the formation of protein aggregates, which masked some of the reactive sulfhydryl groups [1]. Bao et al. [7] suggested that the unavoidable degradation reaction during F-T treatments is also a reason for the decrease in the sulfhydryl content. Cryoprotectants are helpful in inhibiting the unfolding and breaking of protein side-chain structures. Compared with the control samples, the SS group samples had 5.88% higher total sulfhydryl groups, 16.9% higher reactive sulfhydryl groups, and 13.2% lower disulfide bonds after five F-T treatments. Chen et al. [9] demonstrated the inhibitory effect of SS on the decrease in sulfhydryl content and the increase in disulfide bond content. Furthermore, the total and reactive sulfhydryl contents of the NADES group samples reached 5.00 and 2.10 mol/10^5^ g after the fifth F-T cycle, which was higher than those of the control group samples (4.25, 1.54) and the SS group samples (4.50, 1.80) (*p* < 0.05), respectively. Meanwhile, the disulfide bond content of the NADES group samples was lower than those of the other groups (*p* < 0.05). The results indicate that NADES can better protect sulfhydryl groups from freezing damage compared to SS. NADES prepared with citric acid and trehalose has a huge hydrogen bonding network system, and the presence of NADES inhibits the free movement of water and reduces the formation of ice crystals and volume extension, thus reducing the mechanical damage of ice crystals, decreasing the cryo-denaturation and structural deterioration of proteins, which suppresses the exposure of the internal reactive groups, consequently inhibiting the reduction in free sulfhydryl groups and the generation of disulfide bonds [14,19].

#### 3.2.2. Effect of NADES on the MP Secondary Structure of Frozen-Thawed Mirror Carp Surimi

The secondary structure of MP consists of *α*-helix, *β*-sheet, *β*-turn, and random coil, and the folding and binding properties of the secondary structure of MP can be determined and analyzed using circular dichroism [46]. Among the structural constituents, the *α*-helix dominates the secondary structure and is held by the hydrogen bonds (intra-hydrogen bonds of peptide chains) formed by the carbonyl oxygen and the amino hydrogen. The main stabilizing force for the *β*-sheet is the hydrogen bonds between peptide chains, which are particularly critical during protein aggregation [47,48]. In addition, electrostatic interactions between amino acids have a positive effect on maintaining the stability of the secondary structure of MP [2]. To evaluate the effect of the NADES on the MP secondary structure of F-T surimi, the samples that had undergone five F-T treatments were prepared and measured (Table 1). All samples exhibited a decrease in the *α*-helix and *β*-turn contents and an increase in the *β*-sheet and random coil contents. The freezing process produced ice crystals, which caused mechanical damage to the muscle tissue and were accompanied by cold stress effects and solution effects [8]. Reactive oxygen radicals play a crucial role in the process of secondary structure alteration. They alter the microenvironment of MP and accelerate its oxidation reaction. At the same time, the disruption of hydrogen bonds causes the unfolding and aggregation of the protein structure, which is manifested by the transformation of the *α*-helix to the *β*-sheet and random coil [47]. Indeed, the antifreeze agents were effective in maintaining the stability of the MP secondary structure. This can be confirmed by the higher *α*-helix and *β*-turn contents and the lower *β*-sheet and random coil contents in the SS and NADES group samples than in the control group samples for the same number of F-T cycles. SS can bind to polar residues and avoid aggregation reactions between proteins [8]. A NADES binds to proteins to prevent protein uptake on the ice surface, and its interaction with lipid bilayers protects the integrity of the cell membrane (the presence of trehalose) [44]. Thus, a NADES can maximally inhibit the alteration of the MP microenvironment owing to the F-T treatment, which protects the MP secondary structure.

#### 3.2.3. Effect of NADES on the MP Tertiary Structure of Frozen-Thawed Mirror Carp Surimi

The tertiary structure information of MP can be obtained using fluorescence spectroscopy, capturing the chromophores. In the case of muscle proteins, there are three naturally occurring chromogenic groups responsible for fluorescence: phenylalanine (Phe), tryptophan (Trp), and tyrosine (Tyr). However, owing to the low fluorescence quantum yield of Tyr and Phe in proteins, they are rarely used as fluorescent probes [46]. Therefore, ANS extrinsic fluorescent dyes and Trp fluorescent probes were used to analyze the changes in the tertiary structure of MP after multiple F-T treatments. The fluorescence intensity (FI) and the wavelength of maximum fluorescence emission (λmax) of the samples can be used to characterize changes in the tertiary structure of MP by the reflection of tryptophan (Trp) residues against the polarity of the microenvironment [23].

The FI of all samples continued to decrease with increasing F-T cycles (Table 1). This phenomenon occurs due to the disruption of the MP microenvironment by ice crystals. The Trp residues of the indole side chain were originally located in the core of the protein (hydrophobic environment), with the breakdown of homeostasis by ice crystals, the unfolding of the indole side chain, and the exposure of Trp residues (hydrophilic environment) inducing its freezing denaturation. In addition, the change in MP conformation (unfolding) and the formation of an aggregation increased the steric hindrance, which is also a reason for the decrease in the FI of all samples [23]. The λmax, as another index to evaluate the tertiary structure of MP, mainly reflects the microenvironment in which the MP is located. After one F-T treatment, the λmax of the control sample increased from 332.4 nm to 333.0 nm (redshift). This phenomenon indicates a hydrophobic-to-hydrophilic transition of the microenvironment in which the Trp residues are located. The slight blueshift of λmax after three F-T cycles may be due to protein refolding and aggregation, making the microenvironment of Trp residues more hydrophobic [23]. In comparison, the higher FI and the less-fluctuating λmax in the SS and NADES groups indicate their protective effect on the MP tertiary structure. Among all the samples with five F-T treatments, the NADES group samples had the highest FI, exhibiting the strongest stabilizing effect on the MP tertiary structure of the F-T surimi. This is consistent with the results reported in Section 3.2.1.

**Table 1:** Effect of NADES on the MP secondary structure content (%) and tertiary structure of frozen-thawed surimi.

F-T Cycles	Samples	Secondary Structure Content (%)				Tertiary Structure	
		*α*-Helix	*β*-Sheet	*β*-Turn	Random Coil	λ_max_ (nm)	FI (A.U.)
0	Control	60.5 ± 0.6 ^Aa^	17.0 ± 0.5 ^Ad^	9.30 ± 0.25 ^Aa^	13.2 ± 0.4 ^Ad^	332.4 ± 0.3 ^Ab^	1100 ± 5 ^Aa^
	SS	60.8 ± 0.5 ^Aa^	17.2 ± 0.6 ^Ac^	9.25 ± 0.20 ^Aa^	12.8 ± 0.1 ^Ad^	332.3 ± 0.2 ^Aa^	1078 ± 12 ^Aa^
	NADES	60.6 ± 0.6 ^Aa^	17.1 ± 0.4 ^Ac^	9.35 ± 0.30 ^Aa^	13.0 ± 0.7 ^Ad^	332.2 ± 0.2 ^Ac^	1085 ± 20 ^Aa^
1	Control	59.5 ± 0.5 ^Ab^	18.0 ± 0.3 ^Ac^	8.52 ± 0.17 ^Ab^	14.0 ± 0.4 ^Ac^	333.0 ± 0.2 ^Aa^	1050 ± 15 ^Ab^
	SS	59.3 ± 0.3 ^Ab^	17.8 ± 0.4 ^Abc^	8.50 ± 0.35 ^Ab^	14.4 ± 0.3 ^Ac^	332.6 ± 0.3 ^Aa^	1065 ± 8 ^Aa^
	NADES	59.5 ± 0.3 ^Ab^	17.5 ± 0.3 ^Ac^	8.65 ± 0.35 ^Ab^	14.4 ± 0.3 ^Ac^	333.4 ± 0.1 ^Aa^	1060 ± 10 ^Aa^
3	Control	56.3 ± 0.3 ^Bc^	19.5 ± 0.5 ^Ab^	7.45 ± 0.20 ^Ac^	16.8 ± 0.1 ^Ab^	331.6 ± 0.2 ^Ac^	903.0 ± 17.0 ^Bc^
	SS	57.0 ± 0.5 ^ABc^	18.5 ± 0.5 ^Bb^	7.74 ± 0.24 ^Ac^	16.8 ± 0.2 ^Ab^	331.9 ± 0.2 ^Ab^	982.0 ± 12.1 ^Ab^
	NADES	57.5 ± 0.5 ^Ac^	18.3 ± 0.3 ^Bb^	7.80 ± 0.30 ^Ac^	16.4 ± 0.5 ^Ab^	332.9 ± 0.2 ^Bb^	1001 ± 9 ^Ab^
5	Control	53.2 ± 0.5 ^Cd^	21.0 ± 0.4 ^Aa^	5.30 ± 0.15 ^Cd^	20.5 ± 0.3 ^Aa^	331.2 ± 0.2 ^Cc^	774.2 ± 16.3 ^Cd^
	SS	54.5 ± 0.4 ^Bd^	20.0 ± 0.4 ^Ba^	6.15 ± 0.35 ^Bd^	19.4 ± 0.3 ^Ba^	331.7 ± 0.1 ^Bb^	880.4 ± 10.2 ^Bc^
	NADES	55.5 ± 0.3 ^Ad^	19.0 ± 0.4 ^Ca^	6.95 ± 0.20 ^Ad^	18.6 ± 0.2 ^Ca^	332.4 ± 0.1 ^Ac^	924.4 ± 4.6 ^Ac^

The means at the same freeze–thaw cycles with different uppercase letters (A–C) differ significantly (*p* < 0.05); the means at the same NADES concentration with different lowercase letters (a–d) differ significantly (*p* < 0.05).

#### 3.2.4. Effect of NADES on the MP Thermal Stability of Frozen-Thawed Mirror Carp Surimi

**Figure 3:**
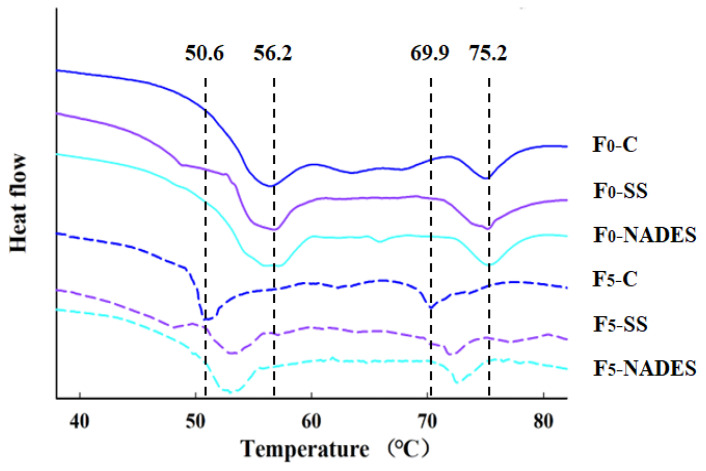
Effect of NADES on the thermal stability of MP from frozen-thawed surimi.

Structural changes in proteins caused by freezing are usually coupled with changes in the thermal stability of proteins, which involve the rupture of covalent and non-covalent bonds [49]. The thermal stability of MP is expressed by the transition temperature (Tmax) and enthalpy (Δ*H*) in the DSC. As shown in Figure 3 and Table 2, *T*_max1_ and *T*_max2_ appeared in the regions of 50.6~56.2 °C and 69.9~75.2 °C, with the end of the denaturation of myosin and actin of the samples, respectively. After five F-T treatments, Tmax1 decreased by 5.6, 3.3, and 3.3 °C, and Tmax2 decreased by 5.3, 3.3, and 2.3 °C for the three groups (control, SS, and NADES) of samples, respectively, indicating that the F-T treatments caused a decrease in the thermal stability of the surimi proteins. This may be caused by the disruption of the protein structure by ice crystals during the F-T process, resulting in the disruption of the overall structure of myosin and actin to form a small subunit during processing [26]. In addition, ice crystals formed during freezing can damage the protein’s secondary structure, leading to a decrease in protein thermal stability and an increase in protein aggregation [50]. It is noteworthy that the *T*_max_ of the NADES group was higher than that of the control and SS groups at the same F-T cycle.

During the F-T process, the trend of Δ*H* in MP is consistent with the change of *T*_max_. The decrease in Δ*H* is due to the growth of ice crystals and recrystallization, as well as the protein denaturation during the freezing and thawing process destroying the MP structure [26]. Additionally, large ice crystals can break the intermolecular hydrogen bonds of proteins, which may cause the denaturation of myosin and the dissociation of myosin subunits or the formation of ROS, ultimately leading to a decrease in the thermal stability of proteins [49]. The ΔH in the NADES group was higher than the other two groups, probably because NADES could effectively inhibit the growth of ice crystals, which reduced the protein damage and protein denaturation, thereby inhibiting the reduction in Δ*H* in the samples.

**Table 2:** Effect of NADES on the maximum transition temperature (*T*_max_) and denaturation enthalpy (Δ*H*) of myosin (peak I) and actin (peak II) of mirror carp surimi induced by freeze–thaw cycles.

F-T Cycles	Samples	*T*_max1_ (°C)	*T*_max2_ (°C)	Δ*H*_1_ (J/g)	Δ*H*_2_ (J/g)
0	Control	56.2 ± 0.2 ^Aa^	75.2 ± 0.1 ^Aa^	1.154 ± 0.007 ^Aa^	0.362 ± 0.004 ^Aa^
	SS	56.3 ± 0.2 ^Aa^	75.3 ± 0.1 ^Aa^	1.155 ± 0.010 ^Aa^	0.370 ± 0.005 ^Aa^
	NADES	56.4 ± 0.1 ^Aa^	75.2 ± 0.1 ^Aa^	1.162 ± 0.006 ^Aa^	0.367 ± 0.005 ^Aa^
5	Control	50.6 ± 0.3 ^Bb^	69.9 ± 0.3 ^Cb^	0.680 ± 0.010 ^Bb^	0.227 ± 0.003 ^Cb^
	SS	53.0 ± 0.3 ^Ab^	72.0 ± 0.2 ^Bb^	0.731 ± 0.005 ^Ab^	0.245 ± 0.003 ^Bb^
	NADES	53.1 ± 0.2 ^Ab^	72.9 ± 0.3 ^Ab^	0.738 ± 0.003 ^Ab^	0.255 ± 0.005 ^Ab^

The means at the same freeze–thaw cycle with different uppercase letters (A–C) differ significantly (*p* < 0.05); the means at the same NADES concentration with different lowercase letters (a–b) differ significantly (*p* < 0.05).

## 4. Conclusions

**Figure 4:**
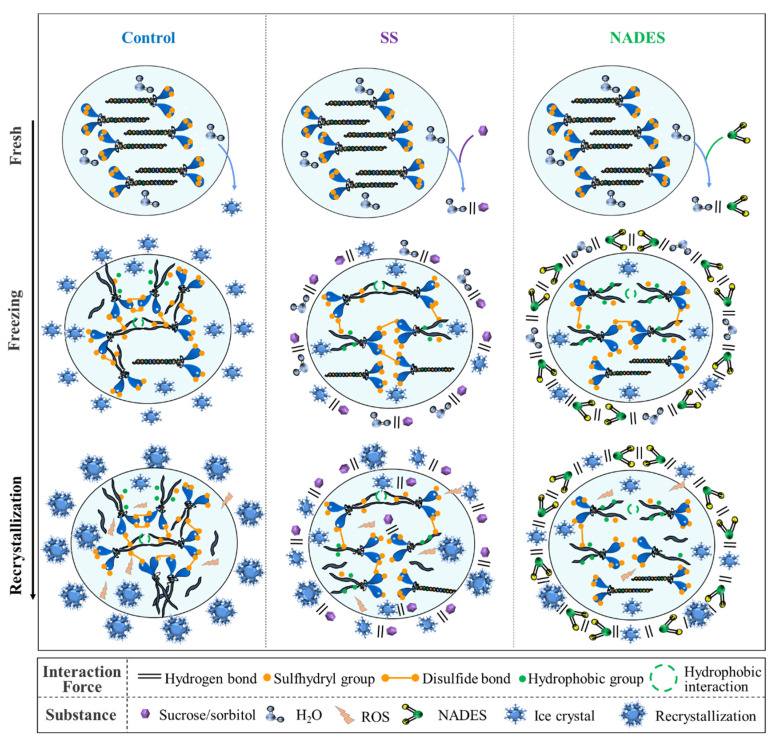
The proposed mechanisms of the NADES-supported cryopreservation.

During the freezing process, the formation of extracellular ice crystals and the occurrence of recrystallization caused osmotic stress and mechanical damage, promoting the accumulation of ROS, which induced protein oxidative denaturation and the structural unfolding of myosin, manifested by an increase in disulfide bonds and hydrophobic interactions and the formation of irregular aggregation (Figure 4). SS could inhibit the growth of ice crystals by binding to ice planes through hydrogen bonding. Meanwhile, SS contains hydroxyl groups that can bind to the polar residues of proteins to saturate the proteins and avoid the formation of aggregation between proteins [8]. The strong hydrogen-bonding network of NADES could effectively trap water and inhibit the flow of water to the ice crystal plane, thus reducing the generation of ice crystals and the occurrence of recrystallization. Ultimately, NADES was superior to SS in reducing the mechanical damage from ice crystals. Meanwhile, NADES samples exhibited lower oxidative denaturation and a more ordered protein structure. This is due to the fact that the immense hydrogen bonding network system of NADES reduces the mechanical damage of ice crystals by decreasing their generation and growth, which inhibits the accumulation of ROS, thus inhibiting the low-temperature denaturation and structural deterioration of proteins, and consequently decreasing the exposure of internal reactive groups, which inhibits the generation of disulfide bonds and carbonyl groups, thus improving the antioxidant capacity of surimi [18,19].

Multiple F-T treatments produced large and unevenly distributed ice crystals, which can puncture the muscle tissue. Subsequently, the release of reactive oxygen radicals and proteases accelerates protein oxidation. At the same time, the unfolding of the MP structure, the formation of aggregates, and changes in the ionic strength of the microenvironment increased the degree of freezing denaturation of the MP. However, the addition of NADES could improve the structural stability and thermal stability while reducing the degree of oxidative denaturation of MP. After confirming the excellent cryoprotective effect of NADES on the F-T surimi MP structure, oxidation, and denaturation, further work can be focused on exploring the effects of NADES on MP functional properties, such as gelation, emulsification, and rheological properties. In addition, the cryoprotective effect of NADES on surimi products needs to be further investigated. In this study, the mechanism of NADES in inhibiting the quality deterioration of frozen-thawed surimi was analyzed from multiple perspectives (oxidative denaturation and structural alteration) at the protein molecular level, which provided a strong guarantee for the application of NADES in the form of antifreeze in the preservation of surimi, provided a theoretical basis for the development and application of novel and green antifreeze, and provided new insights and ideas in improving the quality of frozen freshwater surimi.

## Data Availability

Data is contained within the article.
